# Identification of a potential miRNA–mRNA regulatory network for ischemic stroke by using bioinformatics methods: a retrospective study based on the Gene Expression Omnibus database

**DOI:** 10.3389/fimmu.2025.1467865

**Published:** 2025-04-14

**Authors:** Zhaoying Chen, Xiaodan Zhang, Xiangjun Qi, Jiyuan Zheng, Niancai He, Bohui Zheng, Nan Zhong, Chengcheng Ji, Yulan Jin, Hu Yu, Weinv Fan, Guoming Chen

**Affiliations:** ^1^ The Department of Neurology, Ningbo No.2 Hospital, Ningbo, China; ^2^ Department of Emergency Medicine, The University of Hong Kong, Hong Kong, Hong Kong SAR, China; ^3^ The First Clinical College, Guangzhou University of Chinese Medicine, Guangzhou, China; ^4^ The Fifth Clinical College, Guangzhou University of Chinese Medicine, Guangzhou, China; ^5^ Clinical Medical College of Acupuncture-Moxibustion and Rehabilitation, Guangzhou University of Chinese Medicine, Guangzhou, China; ^6^ The University of Edinburgh, Edinburgh, United Kingdom; ^7^ School of Medicine, Shaoxing University, Shaoxing, China; ^8^ Clinical Laboratory, Ningbo No.2 Hospital, Ningbo, China; ^9^ School of Chinese Medicine, Li Ka Shing Faculty of Medicine, The University of Hong Kong, Hong Kong, Hong Kong SAR, China

**Keywords:** miRNA–mRNA, ischemic stroke, bioinformatics, Gene Expression Omnibus, clinical sample study

## Abstract

**Background:**

Ischemic stroke (IS), a leading cause of disability and death worldwide, lacks effective biomarkers for early diagnosis and therapeutic intervention. This study aims to explore the potential miRNA–mRNA regulatory network in IS using clinical samples and bioinformatics methods, providing insights into its pathophysiology and identifying novel biomarkers.

**Methods:**

We analyzed plasma samples from IS patients and controls collected at Ningbo No. 2 Hospital between May 2022 and February 2023, alongside data from the Gene Expression Omnibus (GEO) database. Bioinformatics analyses, including differential expression analysis and machine learning algorithms, were employed to identify key miRNAs and their target mRNAs. The findings were validated using four-dimensional data-independent acquisition (4D-DIA) quantitative proteomics.

**Results:**

Our analysis revealed differentially expressed miRNAs and mRNAs in IS patients compared to controls. We constructed a potential miRNA–mRNA regulatory network and confirmed the differential expression of proteins associated with this network by proteomic validation, suggesting that they play a role in IS pathophysiology. The results of data analysis and clinical sample validation emphasized Integrin alpha M (ITGAM) as a key gene associated with IS. In addition, ROC curve analysis reflected the good performance of ITGAM as a potential biomarker for the diagnosis of IS and for differentiating between early- and late-onset stroke. The area under curve (AUC) of ITGAM in diagnosing IS was 0.750, and the AUC of ITGAM in distinguishing early-onset stroke from late-onset stroke was 0.759, with a sensitivity of 93.8%.

**Conclusion:**

This study identifies a novel miRNA–mRNA regulatory network in IS, offering potential biomarkers for diagnosis and targets for therapeutic intervention. Our findings bridge the gap between clinical observations and molecular mechanisms, paving the way for improved IS management.

## 1 Introduction

Ischemic stroke (IS) is the leading cause of mortality and disability worldwide (1); its incidence is rising due to the aging population (2, 3). The cost of stroke is also substantial, and most of the costs are for the care of the disability of patients after stroke (4, 5). Improving the functional outcome of IS even modestly would significantly decrease the disease and economic burden of IS (6). The only effective treatment for IS is mechanical thrombectomy within 4.5 h of symptom onset and intravenous thrombolysis within 24 h in selected patients (7, 8). However, thrombolysis and thrombectomy are only applicable to a limited number of IS patients due to their limited treatment window (9). Additional agents are needed to improve the outcomes of IS, especially for those patients not eligible for thrombolysis or thrombectomy, or with no access to stroke unit care (9). Research into miRNAs shows promise for stroke therapy, with profiling of miRNA and mRNA revealing critical genes and miRNAs linked to the condition (11, 12). These molecular biomarkers not only aid in understanding the molecular mechanisms of the condition but may also serve as potential diagnostic and prognostic biomarkers. miRNA-181b regulates signaling pathways related to neural repair by targeting PirB, adding a new dimension to the recovery mechanisms for IS. The upregulation of miRNA-150-5p and miR-181b-5p in the blood after cerebral hemorrhage suggests their potential role as biomarkers in differentiating hemorrhagic from ischemic strokes (13). Therefore, this study hopes to uncover potential treatments for IS by further investigating the mechanisms of the miRNA–mRNA regulatory network.

miRNA and mRNA have been validated as potential targets for the treatment of IS; however, they have not yet been jointly used as biomarkers in clinical practice to analyze their effectiveness, and there is still a significant gap in clinical data in this area. Zhang et al. (14) through ceRNA network analysis revealed potential key miRNAs and target genes in coronavirus disease 2019 (COVID-19)-related chronic obstructive pulmonary disease. Ming-Xi Zhu et al. (14) through findings strongly posit the differentially expressed miRNAs as potential biomarkers to improve stroke diagnosis and prognosis. Kaiser et al. (15) have identified four microRNAs (miRNAs) that are significantly dysregulated in patients with ischemic stroke (IS). These miRNAs, namely, has-miR-4656, has-miR-432, has-miR-503, and has-miR-74-3p, have shown potential as treatment targets for IS. Understanding the roles and functions of miRNAs can provide valuable insights into the development of therapeutic strategies for IS. The research in this field is of great significance, with good clinical benefits and high clinical value (15). However, many previous studies have only focused on identifying diagnostic biomarkers for stroke and have not been successfully applied in clinical practice. The discovery by Xu et al. (16) emphasizes the role of lncRNAs as multi-level regulatory factors in the complex network of post-stroke mechanisms. The analysis of Zhang et al. (17) determined that CDK5R1 RGS2 and NSF are potential diagnostic biomarkers for IS, among others. This may be attributed to factors such as high difficulty and time-consuming clinical translation, limited technical methods, and conditions. At present, most studies have only focused on animal experiments. However, due to significant differences in real pathology and physiology between IS and stroke animal models, further extensive research is needed to confirm whether the miRNA targets currently being sought can be successfully applied in clinical practice (18–20). There is a lack of multicenter research and multi-omics combination research, resulting in poor consistency of research methods and results worldwide (21).

Compared with previous studies, our research incorporates real clinical samples and explores potential miRNA–mRNA regulatory networks in IS through bioinformatics analysis of clinical data results and GEO data. We used clinical specimens from exploration to validation, and combined miRNAs from the database with proteomics from our clinical specimens (22). By combining transcriptomics and proteomics, we validated the targets using real clinical studies, increasing the persuasiveness of our research in terms of depth and breadth. This study constructed a new potential miRNA–mRNA regulatory network, which was validated multidimensional through bioinformatics analysis, machine learning algorithms, and proteomics, providing a new perspective for understanding the pathophysiology of IS. Moreover, we will conduct exploratory analysis on clinical samples from the acute phase within 24 h of onset, which is a lack of biomarker acute phase data in the past. This will help explore biomarkers and therapeutic targets in the acute phase (19). We hope that this study can provide more treatment options for ischemic stroke, and the discovered miRNA–mRNA regulatory network and key genes can provide potential targets for new drug development, promoting the development of IS treatment drugs. At the same time, the expression level of ITGAM may be used to evaluate the prognosis of patients, hoping to help doctors formulate more comprehensive and reasonable rehabilitation plans. In health checkups or high-risk populations, ITGAM can be used as a screening indicator to detect potential stroke risks early and provide preventive interventions. Through these applications, our research results are expected to significantly improve the diagnosis and treatment of IS, bringing good news to IS patients and promoting progress in clinical practice.

## 2 Materials and methods

### 2.1 Gathering of primary data

**Figure 1:**
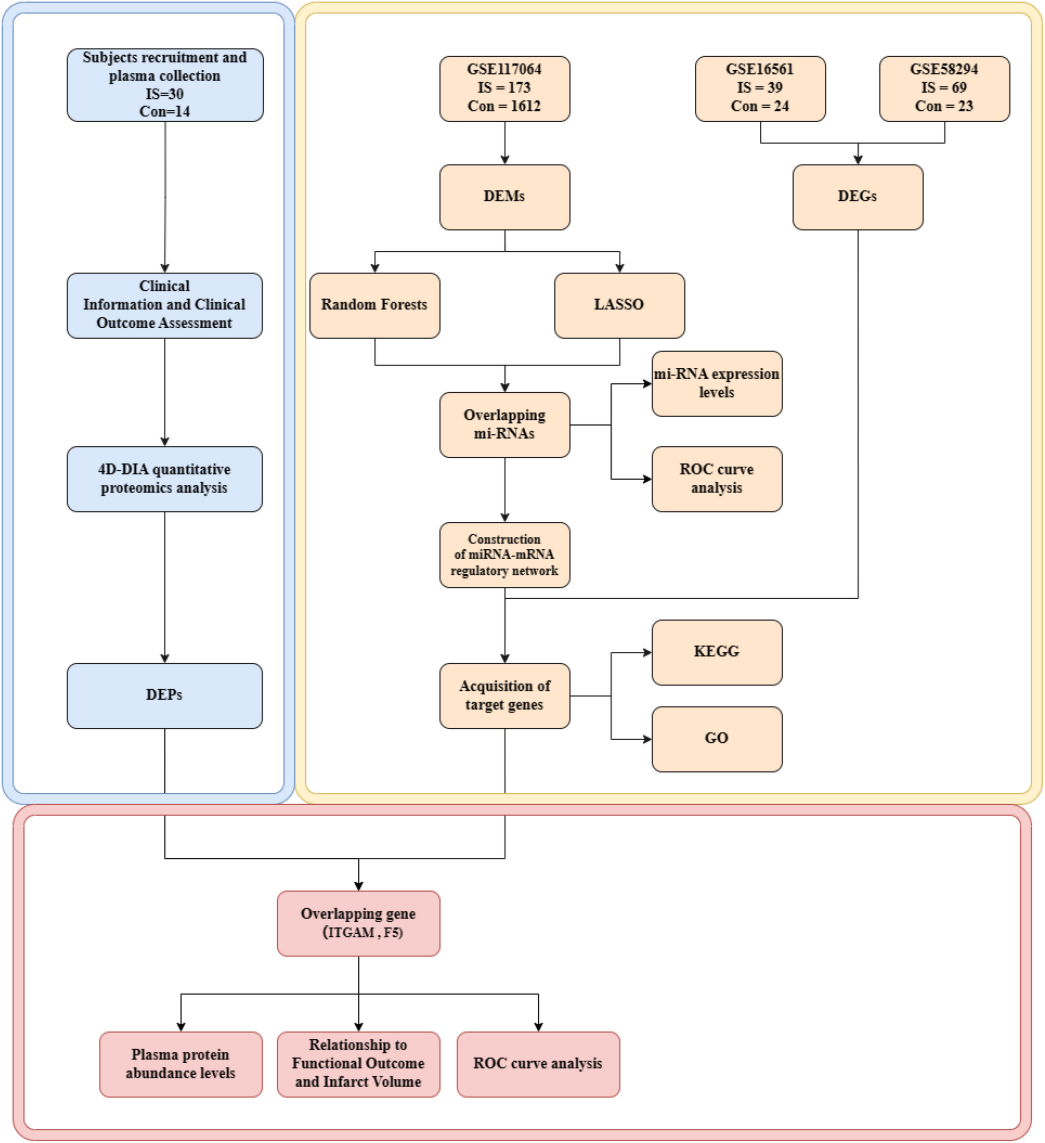
Flow chart of integrated analysis. IS, ischemic strokes; DEMs, differentially expressed miRNAs; DEGs, differentially expressed genes; DEPs, differentially expressed proteins; KEGG, Kyoto Encyclopedia of Genes and Genomes; GO, Gene Ontology.

We were provided with one non-coding miRNA dataset (GSE117064) and two mRNA microarray datasets (GSE16561 and GSE58294) from the Gene Expression Omnibus (GEO) database (https://www.ncbi.nlm.nih.gov/geo/). The GSE117064 dataset includes serum miRNA samples from 173 IS patients and 1,612 non-IS patients using the GPL21263 3D-Gene Human miRNA V21_1.0.0 array. This GSE16561 dataset includes blood RNA samples from 39 IS patients and 24 controls using GPL6883 Illumina HumanRef-8 v3.0 expression bead chip arrays. The GSE58294 dataset includes blood RNA samples from 69 IS patients and 23 controls using the GPL570 [HG-U133_Plus_2]Affymetrix Human Genome U133 Plus 2.0 Array (**Figure 1**).

### 2.2 Data preprocessing and differential expression screening

Using the “GEOquery” package for the R platform, download the miRNA and mRNA data, then import them into the R statistical environment. The R programming language was used for data preprocessing, which included missing data removal, platform annotation files, clinical information, and expression substrate extraction. GEO database platform annotation data were retrieved, and microarray probe IDs were translated to gene symbols. Differential expression analysis was performed by the “limma” package, which compares normal samples with samples from IS patients to obtain differentially expressed genes (DEGs) and differentially expressed miRNAs (DEMs). Critical values for DEMs recognition were adj. p-value<0.05 and |(log2FC)|≥ 1. The key values for DEGs recognition were adj. p-value<0.05 and |(log2FC)|≥ 0.584963.Volcano plots showing differentially expressed miRNAs and mRNAs were generated using the R software “ggplot2” V3.3.5 package.

### 2.3 Selection of miRNA features using machine learning

We employed two machine learning algorithms, namely, Random Forests and LASSO regression analysis, to identify critical genes from differentially expressed miRNAs. Fitting a generalized linear model with variable selection and complexity adjustment was the process of performing LASSO regression analysis. The most pertinent characteristics were expertly found by LASSO regression analysis utilizing the “glmnet” software and 10-fold cross-validation of the penalty settings. In addition, we estimated the prediction efficacy of miRNAs and evaluated their significance using the Random Forest technique via the R package “randomforest.” The Random Forest approach computes the average error rate of candidate center genes to estimate the ideal number of variables. Next, we computed the error rate for every tree ranging from 1 to 500 and identified the ideal number of variables by analyzing the lowest error rate. We built a random forest tree model after figuring out the aforementioned parameters. In the end, we calculated the feature importance score and importance ranking of every putative center gene to choose the right one. The intersecting genes of these two machine-learning algorithms are hub genes for IS patients.

### 2.4 Construction of miRNA–mRNA regulatory network

The target genes of the hub genes were predicted by the miRWalk database (24) (http://mirwalk.umm.uni-heidelberg.de). To produce prediction findings, the miRWalk database primarily collects information from 13 current miRNA–mRNA regulatory linkage databases (such as TargetScan, miRDB, miRTarBase, and TarPmiR). By using Venn diagrams to integrate the prediction findings of hub gene target mRNAs with the overlapping genes of DEGs from the GSE16561 and GSE58294 datasets, networks were constructed by sieve-selecting DEGs and hub genes with regulatory links. With Cytoscape 3.1.0, the miRNA–mRNA regulatory networks were constructed and illustrated.

### 2.5 Functional enrichment analysis

We used the “clusterProfiler” package in the R platform to analyze 21 target genes in the miRNA–mRNA regulatory network to evaluate the biological activities of hub gene target mRNAs. We did Gene Ontology (GO) classification (http://geneontology.org/) and the Kyoto Encyclopaedia of Genes and Genomes (KEGG) pathway (https://www.genome.jp/kegg/) studies on these target genes.

### 2.6 Subjects recruitment and plasma collection

Consecutive IS patients were recruited from the Emergency Department (ED) of Ningbo No. 2 Hospital from May 2022 to February 2023. The inclusion criteria were 1) acute IS patients, diagnosed by neurologists combining the clinical and diffusion-weighted imaging (DWI) lesion on MRI or a new lesion on a delayed CT scan; 2) within 24 h of symptom onset; 3) age ≥50 years old; and 4) informed consent. The exclusion criteria were 1) patients who received thrombolysis or thrombectomy before enrolment; 2) active malignant disease, hematological disease, inflammatory or infectious diseases; 3) renal or liver failure; 4) tumors; and 5) surgery within the past 3 months.

Controls were recruited from the volunteers from the Physical Examination Center and out of patients of Ningbo No. 2 hospital from May 2022 to February 2023. Subjects included in this study 1) were aged ≥18 years old and 2) signed informed consent. We excluded those with 1) ischemic stroke and intracerebral hemorrhage; 2) active malignant disease, hematological disease, and inflammatory or infectious diseases; 3) renal or liver failure; 4) tumors; and 5) surgery within the past 3 months.

Blood samples were collected from patients within 24 h after stroke onset as soon as they arrived at ED and before any therapies. Blood samples were also drawn from controls. Approximately 5 ml peripheral blood sample was drawn in an ethylene diamine tetra-acetic acid (EDTA) bottle from subjects. The blood samples were then centrifuged at 3,500 rpm for 10 min at 4°C, and plasma was collected and stored at −80°C until required. This study was performed in line with the principles of the Declaration of Helsinki. Approval was granted by the ethics committee of the Ningbo No. 2 Hospital (No: YJ-NBEY-KY-2023-099-01). Informed consent was obtained from all individual participants included in the study.

### 2.7 Clinical information and clinical outcome assessment

We obtained basic demographic data and clinical history including vascular risk factors, stroke subtypes, reperfusion therapy, stroke onset time, and blood sampling time. Vascular risk factors included hypertension (systolic blood pressure >140 mmHg or diastolic blood pressure >90 mmHg or on antihypertensive medication), diabetes mellitus (fasting blood sugar >7.0 mmol/L or hemoglobin A1c >6.5% or on glucose-lowering medication), hyperlipidemia, and smoking and drinking status. Stroke etiology was determined by Trial of Org 10172 in Acute Stroke Treatment classification as (1) large artery atherosclerosis (LAA), (2) cardioembolic infarction (CE), and (3) small vessel occlusion (SVO). Patients with other determined etiology and undetermined etiology were excluded.

An independent neurologist assessed the patient’s functional status using the National Institutes of Health Stroke Scale (NIHSS) on admission and discharge and modified Rankin’s score (mRS) at discharge and 3 months.

### 2.8 Statistical analysis

Data were statistically analyzed using R 4.0.3 software. Measurement data were tested for normality using the Shapiro normality test. Normally distributed data were expressed as the mean ± standard deviation (SD), and comparisons between groups were made using the independent samples t-test. Non-normally distributed data were expressed as median (interquartile range), and between-group comparisons were made using the Mann–Whitney U-test. Count data were expressed as the number of instances (percentage), and between-group comparisons were made using Fisher’s exact test and chi-square test. Generated subject work characteristics [receiver operating characteristics (ROC)] curves were plotted to assess the biomarkers’ efficacy. Statistical differences between the normalized expressions of the screened genes were assessed using comparative t-tests. p-values of <0.05 were considered statistically significant.

## 3 Results

### 3.1 Identification of DEMs

**Figure 2:**
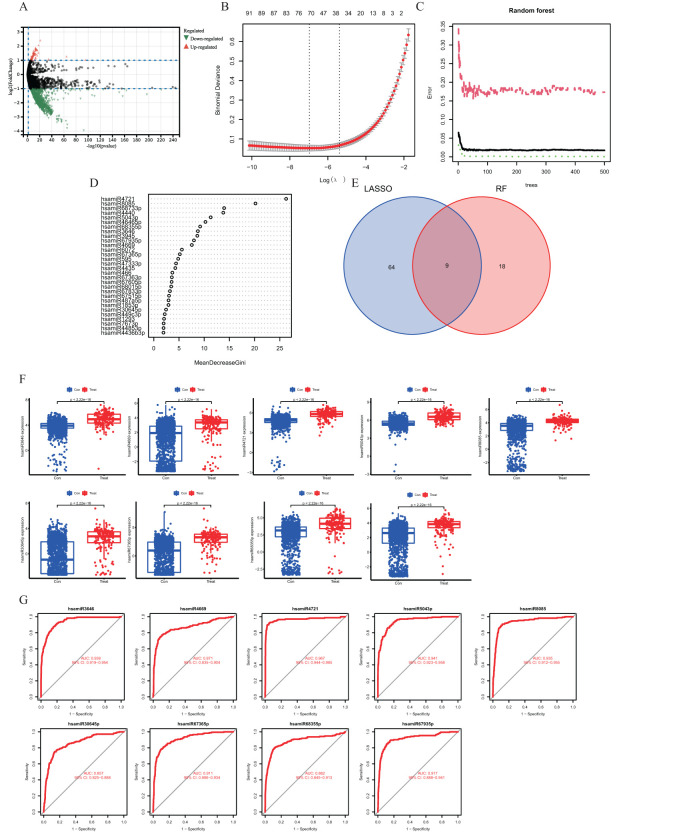
**(A)** Volcano diagram showing differences in gene expression of miRNAs. Orange dots indicate upregulated genes, green dots indicate downregulated genes, and gray dots indicate genes with no significant changes. **(B)** Demonstrates the cross-validation error of the LASSO model with different regularization parameters. The horizontal coordinate is the logarithmic value of the regularization parameter, and the vertical coordinate is the cross-validation error. **(C)** Performance evaluation results of the random forest model. The horizontal coordinate indicates the number of trees in the random forest, and the vertical coordinate indicates the error rate of the model. **(D)** Venn diagram showing the overlap of feature genes selected in the LASSO and random forest models. **(E)** Scatterplot demonstrating the ordering of the top 30 genes in the importance score in the random forest model. **(F)** Box line plot demonstrating the comparison of the expression levels of the characterized genes in the control and IS groups. **(G)** ROC curve demonstrating the performance of the characterized genes in the classification model.

For analysis, we took the miRNA data out of GSE117064. A study was conducted on the expression levels of miRNAs in 1,612 non-IS sera and 173 IS patients. Using |log2FC|≥1 and adj. p-value < 0.05 as criteria, we found 67 upregulated and 756 downregulated miRNAs in the control group compared to the IS patient sample. The volcano plot in **Figure 2** displays the log2FC correlation and the −log miRNA distribution (10) (p-value), indicating that downregulated miRNAs are more significant in log 2FC than upregulated miRNAs (**Figure 2A**).

### 3.2 Hub gene identification by applying the LASSO regression and Random Forest algorithms

**Figure 3:**
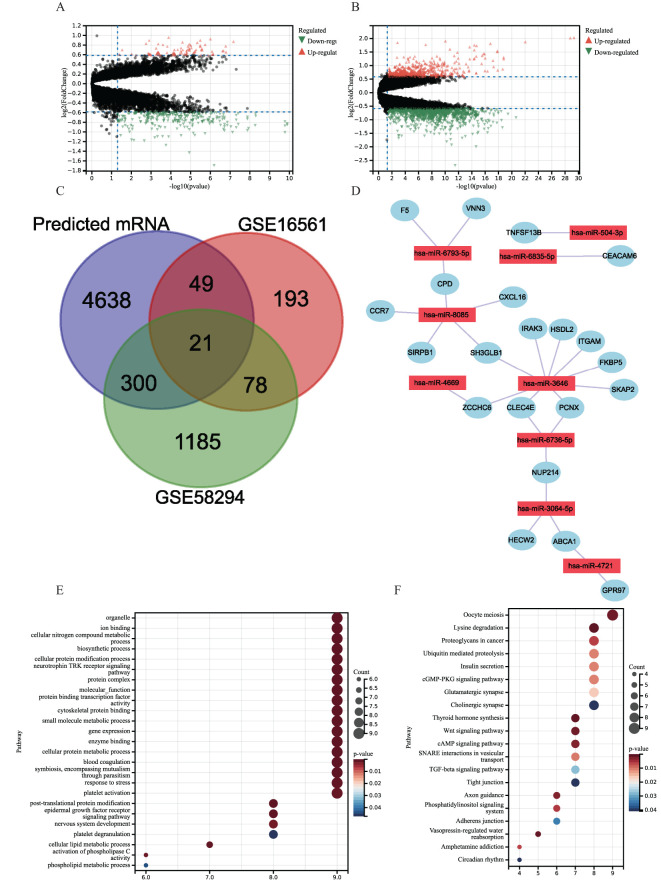
**(A, B)** Volcano plots of DEGs distribution in GSE16561 **(A)** and GSE58294 **(B)**. Orange dots indicate upregulated genes, green dots indicate downregulated genes, and gray dots indicate genes with no significant changes. **(C)** Venn diagram showing overlapping genes of GSE16561 and GSE58294 differential genes with predicted mRNAs. **(D)** miRNA–mRNA Regulatory networks. **(E, F)** Gene Ontology (GO) and Kyoto Encyclopedia of Genes and Genomes (KEGG) pathway enrichment analysis of 21 target genes in miRNA–mRNA regulatory networks. **(E)** GO. **(F)** KEGG.

We employed the Random Forest and LASSO regression machine learning algorithms to find putative hub genes. Using LASSO regression analysis, we were able to identify 73 miRNAs as the most relevant described genes, including hsa-let-7a-5p, hsa-let-7d-5p, hsa-miR-16-5p, hsa-miR-17-5p, hsa-miR-21-5p, hsa-miR-154-5p, hsa-miR-551b-3p, hsa-let-7f-1-3p, hsa-miR-29b-2-5p, and hsa-miR-186-3p (**Figure 2B**). In the Random Forest model, we chose miRNAs with an importance score >2 as feature variables (**Figures 2C, D**), and the Random Forest approach demonstrated a consistent error rate (**Figure 2E**). Using Venn diagram analysis, we were able to identify nine miRNAs as essential hub genes: hsamiR3646, hsamiR4669, hsamiR4721, hsamiR5043, hsamiR8085, hsamiR30645p, hsamiR67365p, hsamiR68355, and hsamiR67935p (**Figure 3D**).

### 3.3 Increased hub gene expression in the serum of IS patients

In comparison to healthy controls, the GSE117064 dataset revealed hub gene greater expression in IS patients, which was all statistically significant (**Figure 2F**).

### 3.4 Diagnostic performance of the characterized hub genes

By ROC curve analysis, we evaluated the diagnostic performance of nine hub genes: hsamiR3646, hsamiR4669, hsamiR4721, hsamiR5043, hsamiR8085, hsamiR30645p, hsamiR67365p, hsamiR68355, and hsamiR67935p. The respective ROC curves yielded area under the curve (AUC) values of 0.938, 0.871, 0.967, 0.941, 0.935, 0.857, 0.911, 0.882, and 0.917 (**Figure 2G**). The AUCs of the nine pivotal genes were >0.7, which demonstrated their potential as diagnostic markers.

### 3.5 Construction of miRNA–mRNA regulatory network

Using the “limma” package, DEGs were extracted from the datasets GSE16561 and GSE58294 and displayed using volcano plots (**Figures 3A, B**). Using the miRWalk platform, we were able to identify 6,317 miRNA–mRNA regulatory pairings in total. A total of 21 genes overlapped with DEGs in the miRNA–mRNA regulatory pairings (**Figure 3C**). Nine hub genes controlled these overlapped genes. Next, a regulatory network was created and shown connecting the nine hub genes and the 21 DEGs (**Figure 3D**). Compared with IS patient samples, CCR7 expression was upregulated in the control group and SKAP2, CPD, FKBP5, VNN3, GPR97, SH3GLB1, PCNX, HECW2, ITGAM, CEACAM6, CLEC4E, ZCCHC6, F5, HSDL2, NUP214, ABCA1, SIRPB1, IRAK3, TNFSF13B, and NXCL16 expression downregulation.

### 3.6 Functional enrichment analysis

A total of 21 target genes underwent enrichment analysis to get more insight into their putative biological roles and signaling cascades. The results showed that the target genes in the GO enrichment analysis were mainly involved in cellular metabolism (e.g., cellular nitrogen compound metabolism, cellular protein cell protein metabolism, and cellular lipid metabolism) and coagulation mechanisms (blood coagulation, platelet activation, and platelet degranulation) (**Figure 3E**). In addition, KEGG enrichment showed that 21 target genes were concentrated in proliferation, intercellular communication, and coagulation-related pathways, such as oocyte meiosis, cGMP-PKG signaling pathway, cAMP signaling pathway, thyroid hormone synthesis, Wnt signaling pathway, and other pathways (**Figure 3F**).

### 3.7 Proteomic profile in IS patients

The study sample size for comparison in the discovery phase, when targeted AUCs of the biomarkers >0.9, was at least 14 per group. Finally, we enrolled 30 IS and 14 health controls. A total of 30 patients with IS attended the ED between June 2022 and February 2023. MRI with DWI data or CT with lesions were available for 28 patients. Infarct volumes were determined by one experienced neurologist who was unaware of the patient’s clinical and laboratory results. The infarct volume was calculated using the ABC/2 method (A and B represent the largest diameter of the infarct and its largest perpendicular diameter, respectively, whereas C represents the thickness of the slices with a visible infarct lesion).

**Figure 4:**
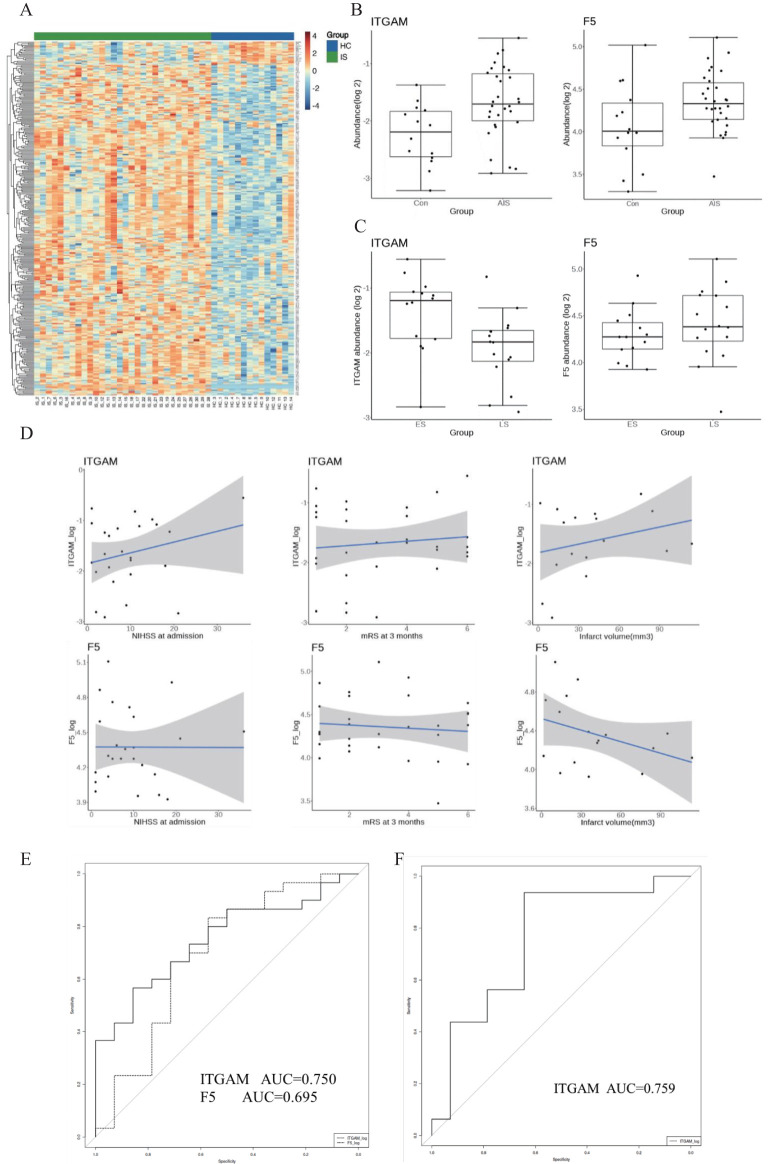
**(A)** Heatmap of the plasma proteomic profiling of 30 IS patients and 14 healthy controls. Red areas represent upregulation, and blue areas represent downregulation. **(B)** Abundance of the two plasma proteins in the IS group and the control group. **(C)** Abundance of the two plasma proteins in the ES group and LS group. ES, early-onset stroke within 4.5 h. LS, late-onset stroke within 4.5–24 h. **(D)** Association with ITGAM, F5 with function outcome and infarct volume in IS patients. **(E)** The diagnostic value of ITGAM and F5 for differentiating IS from controls. **(F)** The diagnostic value of ITGAM in differentiating patients with early-onset stroke from those with late-onset stroke.

Pooled plasma samples of the subjects were analyzed using four-dimensional data-independent acquisition (4D-DIA) quantitative proteomics analysis. Applying the criteria |log2FC|≥ 0.263 and an adj. p-value < 0.05, there were 296 upregulated and 21 downregulated plasma proteins in IS patients compared with controls, as shown in the hierarchical clustering heat map (**Figure 4A**).

F5 and ITGAM were found to overlap in the human plasma proteomic result and the identified 21 targeted genes. ITGAM is known to be involved in the apoptotic signaling pathway and cell–cell adhesion, and F5 is primarily associated with coagulation function. The plasma abundance levels of F5 and ITGAM were significantly higher in the IS patients compared with controls (**Figures 4B, C**, **Tables 1**–**3**).

**Table 1:** Baseline characteristics of patients with IS and healthy control.

Variables	IS (n=30)	Control (n=14)	p
Age,mean (SD), years	70.6 ± 7.68	64.86 ± 6.75	0.021
Gender			0.620
Female, n (%)	7 (23.33%)	5 (35.71%)	
Male, n (%)	23 (76.67%)	9 (64.29%)	
Smoking, n (%)	4 (17.39%)	0 (0%)	1.000
Diabetes mellitus, n (%)	9 (30%)	4 (28.57%)	1.000
Hypertension, n (%)	16 (53.33%)	5 (35.71%)	0.276
Hyperlipidemia, n (%)	3 (10%)	1 (7.14%)	1.000
Stroke onset to sampling time (h)	2.72 (1.52,4.07)	NA	NA
Stroke etiology			
Large-artery atherosclerosis, n (%)	12 (40%)	NA	NA
Cardio-embolism, n (%)	12 (40%)	NA	NA
Small-vessel occlusion, n (%)	6 (20%)	NA	NA
Thrombolysis <4.5 h, n (%)	11 (36.67%)	NA	NA
Thrombectomy <24 h, n (%)	12 (40%)	NA	NA
Stroke volume (mm^3^)	35.7 (64.9)	NA	NA
NIHSS at admission	10 (14)	NA	NA
NIHSS at discharge	3 (4)	NA	NA
mRS at discharge	3 (1)	NA	NA
mRS at 3 months	3 (3)	NA	NA

Data are shown as mean (SD), and median (IQR) for continuous variables, and as percentages for categorical variables.

IS, ischemic stroke; NIHSS, National Institutes of Health Stroke Scale; mRS, modified Rankin Scale; HAMD-24, Hamilton Rating Scale for Depression-24.

**Table 2:** Abundance of the two plasma proteins in the IS group and control group.

	FC	p	Control (n=14)	IS (n=30)	p
ITGAM_log	1.48	0.005	−2.24 ± 0.53	−1.67 ± 0.62	0.005
F5_log	1.22	0.029	4.07 ± 0.48	4.36 ± 0.34	0.029

**Table 3:** Abundance of the two plasma proteins in the ES group and LS group.

	ES (n=14)	LS (n=16)	p
ITGAM_log	−1.38 ± 0.59	−1.93 ± 0.55	0.014
F5_log	4.3 ± 0.27	4.41 ± 0.4	0.372

ITGAM exhibited a positive correlation trend with stroke severity (NIHSS score at admission), 3-month functional outcomes (mRS score at 3 months), and infarct volume in IS patients. In contrast, F5 did not show a significant relationship between stroke severity and 3-month functional outcomes. However, it displayed a negative correlation trend with infarct volume in IS patients (**Figure 4D**).

We further divided the IS group into two subgroups: early-onset IS, which includes cases where the stroke onset to sampling time was within 4.5 h, and late-onset IS, which includes cases where the stroke onset to sampling time was between 4.5 and 24 h. Interestingly, the abundance of F5 showed no significant difference between early and late-onset IS patients, while ITGAM was significantly higher in early early-onset IS group. We further assessed the diagnostic value of the protein biomarkers. The AUC were 0.750 (0.601–0.899) for ITGAM and 0.695 (0.515–0.875) for F5, respectively, for diagnosing patients with IS from controls (**Figure 4E**, **Table 4**). The AUC of ITGAM were >0.7 with a specificity of 0.857, which demonstrated its potential as a diagnostic marker. We further investigated the diagnostic value of ITGAM to distinguish early- from late-onset stroke patients. The AUC value for ITGAM was 0.759 (0.577–0.941), with a sensitivity as high as 93.8% (**Figure 4F**, **Table 4**).

**Table 4:** The diagnostic value of ITGAM and F5 for IS vs. control and ITGAM for early-onset stroke vs. late-onset stroke.

	AUC	p	AUC [95%CI]	Cut point	Sensitivity	Specificity	Accuracy
ITGAM_log	0.750	0.007	[0.601,0.899]	−1.75	0.567	0.857	0.659
F5_log	0.695	0.039	[0.515,0.875]	4.05	0.833	0.571	0.750
ITGAM_log	0.759	0.015	[0.577,0.941]	−1.27	0.938	0.643	0.8

## 4 Discussion

In this study, by identifying 21 key targets of miRNA, we conducted GO and KEGG enrichment analyses. The enriched results revealed that the genes targeted by IS-related miRNAs are primarily involved in regulating immune and inflammatory pathways, such as the cAMP and Wnt signaling pathways. In the process of leukocyte adhesion, the cAMP signaling pathway influences the interaction between leukocytes and vascular endothelial cells by modulating the expression of adhesion molecules on the cell surface, which is critical for leukocyte migration and the onset of inflammatory responses (23). Experimental research has also found that atorvastatin can regulate the pro-inflammatory/anti-inflammatory phenotype switch in murine brain microglia through the Wnt/β-catenin pathway, thereby protecting neonatal rats with ischemic brain injury (24).

Upon data analysis and clinical sample validation, we discovered the heightened expression of Integrin alpha M (ITGAM), targeted by has-miR-3646 in IS samples, especially within 4.5 h of onset. Correlating with clinical features, the expression levels of ITGAM are positively associated with the severity of the stroke, poorer functional outcome, and the extent of infarction, drawing our attention to the potential of has-miR-3646-ITGAM as a therapeutic target for IS.

ITGAM is a membrane surface glycoprotein and a member of the integrin family. Integrins are a class of cell surface receptors that regulate interactions between cells and between cells and the extracellular matrix (ECM) (25). ITGAM, primarily expressed on leukocytes, especially monocytes and neutrophils, mediates various immune and inflammatory responses, including cell adhesion, migration, and phagocytosis (26). It is well known that brain cell injury and death are key pathological features of IS. In the early stages, the activation of microglia can increase the permeability of the blood–brain barrier (BBB), facilitating the adhesion and migration of immune cells (27). Moreover, the various cytokines produced by microglia can increase the expression of adhesion molecules on vascular endothelial cells, such as selectins and integrins, promoting the adhesion of immune cells, particularly neutrophils and monocytes, to the vascular endothelium (28). These adherent immune cells migrate across the endothelium into the brain tissue, participating in the inflammatory response (29). As part of the integrin family, ITGAM may be implicated in post-stroke inflammatory responses, including promoting leukocyte adhesion and migration, thereby exacerbating brain tissue damage (30).

This study has several limitations that should be acknowledged. First, the proteomic sample size was relatively modest, which may constrain the generalizability of the research findings. A larger cohort of patients would be necessary to validate the potential biomarkers and therapeutic targets identified in this study. Second, the miRNA database employed was not composed exclusively of acute ischemic stroke patients, and serum samples were utilized, which may affect the specificity of the miRNA–mRNA regulatory network identified for this particular condition. Third, the results of this study require further validation through prospective clinical trials before they can be applied in a clinical setting. The potential biomarkers and therapeutic targets identified here show promise, but their clinical utility and effectiveness need to be rigorously tested in future studies. In summary, these results suggest that ITGAM is associated with brain cell apoptosis, correlated to stroke severity and unfavorable functional outcomes. ITGAM has the potential to be utilized in clinical practice to specifically confirm AIS and sensitively exclude late-onset stroke patients, selecting suitable AIS patients for early reperfusion therapies.

## Data Availability

The datasets presented in this study can be found in online repositories. The names of the repository/repositories and accession number(s) can be found in the article/supplementary material.
